# Active Bending of Disordered Microtubule Bundles by Kinesin Motors

**DOI:** 10.1021/acsomega.2c04958

**Published:** 2022-11-18

**Authors:** Vahid Nasirimarekani, Smrithika Subramani, Sebastian Herzog, Andrej Vilfan, Isabella Guido

**Affiliations:** † Max Planck Institute for Dynamics and Self-Organization (MPIDS), Am Fassberg 17, 37077Göttingen, Germany; ‡ Department of Physics, University of Wisconsin-Milwaukee, 3135 N Maryland Avenue, Milwaukee, Wisconsin53211, United States; § Department for Computational Neuroscience, Third Institute of Physics − Biophysics, University of Göttingen, Friedrich-Hund-Platz 1, 37077Göttingen, Germany; ∥ Jožef Stefan Institute, Jamova 39, 1000Ljubljana, Slovenia

## Abstract

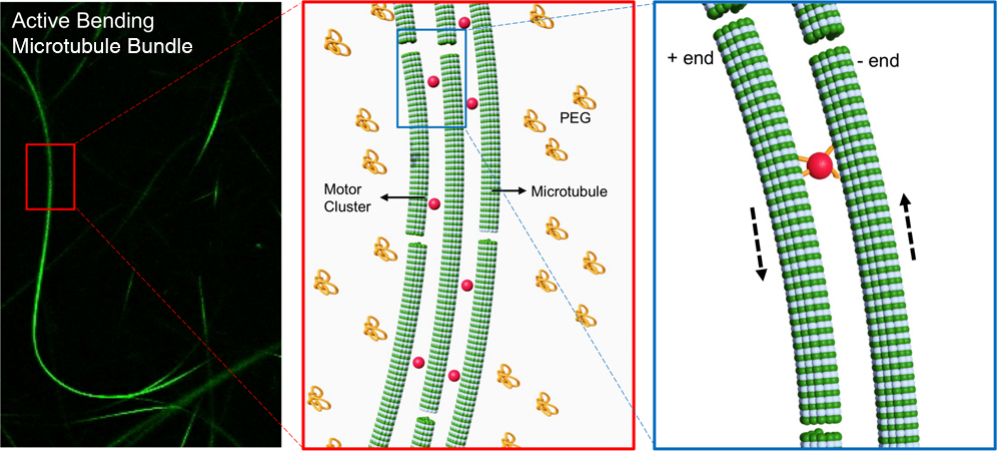

Active networks of biopolymers and motor proteins in vitro self-organize and exhibit dynamic structures on length scales much larger than the interacting individual components of which they consist. How the dynamics is related across the range of length scales is still an open question. Here, we experimentally characterize and quantify the dynamic behavior of isolated microtubule bundles that bend due to the activity of motor proteins. At the motor level, we track and describe the motion features of kinesin-1 clusters stepping within the bending bundles. We find that there is a separation of length scales by at least 1 order of magnitude. At a run length of <1 μm, kinesin-1 activity leads to a bundle curvature in the range of tens of micrometers. We propose that the distribution of microtubule polarity plays a crucial role in the bending dynamics that we observe at both the bundle and motor levels. Our results contribute to the understanding of fundamental principles of vital intracellular processes by disentangling the multiscale dynamics in out-of-equilibrium active networks composed of cytoskeletal elements.

## 1 Introduction

Active bundles of microtubules and motor proteins are involved in many cellular functions such as cell division,^1^ cell migration,^2^ cytoplasmic streaming,^3^ propulsion, and fluid transport driven by cilia and flagella.^4^ In addition to their biological relevance, these networks show remarkable behavior also outside the cells. Studied in vitro, they represent a prime example of active and living matter physics systems.^5^ Dense networks of microtubules and motor proteins that exhibit higher-level self-organization and pattern formation have been intensively studied in the recent past.^6,7^ Many of these setups also involve a depletion agent such as poly(ethylene glycol) (PEG) mixed into a solution of microtubules and kinesin motors arranged as multimeric clusters.^8^ PEG in the bulk aggregates the filaments and forms bundles held together by depletion forces.^9^ Kinesin motors are commonly used in the form of clusters containing several dimeric kinesin molecules and can simultaneously bind at least to two microtubules. Driven by the continuous supply of fuel due to the presence of ATP in solution, they cross-link the filaments and slide them against each other. Namely, when the kinesin clusters link microtubules that are close to each other due to the depletion force, the motors move toward the plus ends of the filaments. In parallel microtubules, the motors can induce beating and wave formation mimicking the function of biological cilia.^10,11^ When bound microtubule pairs have opposite polarity, the kinesin activity generates their extension. In this configuration, the active system exhibits extensile motility, which in hierarchical assemblies provides a basis for a wide range of active nematic phenomena.^8,12−14^ Many experimental studies have investigated the large scale non-equilibrium dynamics of active nematics and how it can be varied by external cues, for instance, by confinement, such as structured liquid interfaces,^15^ hard wall circular boundaries,^16^ curved surfaces of deformable spherical vesicles,^17^ and toroidal droplets,^18^ as well as by radial alignment.^19^ However, how the individual constituents of the active networks behave to generate such dynamics is still an open question.

**Figure 1:**
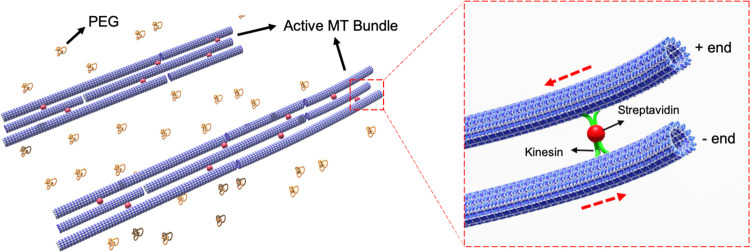
Schematics of the active microtubule bundles and binding of kinesin motor clusters on multiple filaments. The inset view shows the bending mechanism of active microtubule bundle. Red dash arrows show the movement direction of the microtubules due to the antiparallel polarity of the filaments.

In this study we investigate an active network of microtubules and kinesin motor clusters in two different configurations (Figure 1). In the first case, we visualize and analyze individual bundles of depleted microtubules. We characterize their dynamics without considering the interaction with the neighboring bundles in the dense network, but only the active stress generated by the motor clusters. In the second case, we study the motion of the motors within and between the bundles. A recent study quantified the sliding motion both of isolated microtubule pairs and dense active nematics comparing their extension rate.^20^ Here, we show that the bending of microtubule bundles and the activity of the motors that generate it happen at different length scales, highlighting the multiscale dynamics of these hierarchically organized active systems.

## 2 Experimental Section

Here, we studied the dynamics of active networks made of microtubules and motor proteins kinesin-1 organized in clusters at different length scales. For this purpose, we characterized the bending behavior of individual microtubule bundles and the stepping strategy of the motor proteins that induces the bending activity.

### 2.1 Preparation of Active Microtubule–Kinesin Networks

#### 2.1.1 Kinesin Clusters Assembly

The plasmid that codes biotin-labeled kinesin 401 (K401) was a gift from Jeff Gelles (pWC2 – Addgene plasmid #15960; http://n2t.net/addgene: 15960; RRID_Addgene_15960)^21^ and was purified according to previously published protocols.^22,23^ To create active kinesin clusters, multimotor complexes were prepared by mixing 0.2 mg/mL kinesin-1 and 0.1 mg/mL streptavidin (Sigma, S4762) in M2B buffer (M2B: 80 mM PIPES, adjusted to pH = 6.8 using KOH, 1 mM EGTA, 2 mM MgCl_2_) containing 0.9 mM DTT and incubated on ice for 15 min. Four microliters of this mixture were combined with 1% PEG and 2 mM ATP. The final concentration of kinesin in the solution was 17 nM. To maintain a steady ATP concentration for the entire experimental duration, an ATP regeneration system containing 32 mM phosphoenol pyruvate (PEP, Alfa Aesar B20358) and 1.7 μL of pyruvate kinase/lactic dehydrogenase enzymes (PK/LDH, Sigma, P-0294) was incorporated. To reduce photobleaching effects, an oxygen scavenging mix containing 0.2 mg/mL glucose oxidase (Sigma, G2133), 0.05 mg/mL catalase (Sigma, C40), 2.4 mM Trolox (Sigma, 238813), 0.5 mg/mL glucose, and 0.65 mM DTT was included. For experiments with labeled motor clusters, 0.1 mg/mL Cy-3-labeled streptavidin (Sigma, S6402) was used to create the kinesin complexes.

#### 2.1.2 Polymerization of Active Microtubule Bundles

Microtubule bundle polymerization mixtures were prepared by mixing 2.7 mg/mL of unlabeled and labeled porcine brain tubulin (Cytoskeleton, Inc.) (1:5 ratio Hilyte488 labeled:unlabeled tubulin) in M2B with 4 mM MgCl_2_, 5% DMSO, 1 mM GTP, and 1% poly(ethylene glycol) (PEG: MW 20 kDa, Sigma 95172). The polymerization mixture was combined with the kinesin clusters described above, with the inclusion of a high-salt M2B (M2B with 7.8 mM MgCl_2_) containing 7 μM Taxol. The final concentration of tubulin in solution was 8 μM. It was incubated at 37 °C for 45 min, resulting in an active network of microtubule bundles. When required, the polymerized active network samples were diluted 20- to 2000-fold for the visualization of the individual bundles. Afterward, it was pipetted into the experimental chamber and subsequently imaged.

### 2.2 Protein-Repellent Surfaces

Glass coverslips (24 × 60 mm^2^, VWR) were cleaned in a series of steps. They were washed with 100% ethanol and rinsed in deionized water. This was followed by sonication in acetone for 30 min and incubation in ethanol for 10 min at room temperature. The coverslips were then incubated in a 2% Hellmanex III solution (Hellma Analytics) for 2 h, washed extensively in deionized water, and dried using filtered airflow. To functionalize the surfaces, the cleaned coverslips were activated in oxygen plasma (FEMTO, Diener Electronics, Germany) for 30 s at 0.5 mbar and subsequently incubated in 0.1 mg/mL poly(l-lysine)-*graft*-poly(ethylene glycol) (PLL-*g*-PEG) (SuSoS AG, Switzerland) in 10 mM HEPES, pH = 7.4 at room temperature on parafilm. After 2 h, the coverslips were carefully lifted off and the remaining PLL-*g*-PEG solution at the edges was removed.

### 2.3 Sample Chamber Assembly, Imaging, and Tracking

Experimental chambers were prepared by cutting a window of size 8 × 8 mm^2^ on a 10 μm thick double-sided tape (No. 5601, Nitto Denko Corporation, Japan), using it as a spacer between two PLL-*g*-PEG functionalized coverslips facing each other (see Figure S1 for details). The chambers were completely sealed after an equivalent volume (∼2 μL) of the active sample was pipetted into the window. Images of the resulting kinesin cluster–microtubule networks were acquired using an Olympus IX81 inverted fluorescence microscope (Olympus, Japan) with a 63× oil-immersion objective (Olympus, Japan). Samples were excited using a Lumen 200 metal arc lamp (Prior Scientific Instruments), and a series of images were recorded with a Photometrics Cascade II EMCCD camera. The frame size was 960 × 960 pixels, and the images were acquired at a frequency of 1 Hz.

Image acquisition of the diluted bundles was performed using a confocal laser scanning microscope setup (Olympus FluoView 1000) with a 60× UPlanSApo objective. The frame size was 512 × 512 pixels. The bundle contours in these images were tracked using JFilament^24^ and Fiji,^25^ where the segmentation routine was developed in house and the tracking was done with TrackMate.^26^ The segmentation consists of several steps as shown in Algorithm 1 to extract masks from the images which remove everything except the motors to be tracked. The application of Algorithm 1 provides segmented images which are then directly used as input in TrackMate.^26^ In TrackMate, the parameters for the maximal linking distance were set to 2.0 pixel, with a maximal gap closing distance of 2.0 pixel and no gap closing. The implementation can be found in the Supporting Information and is also publicly available.

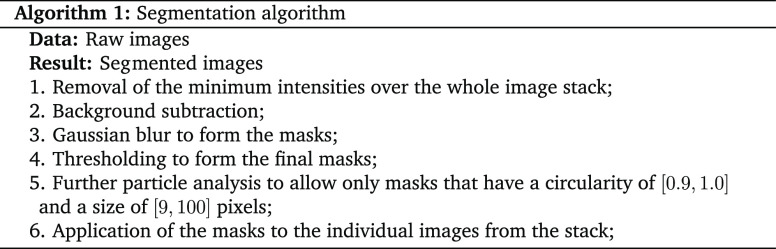



### 2.4 Shape Analysis

**Figure 2:**
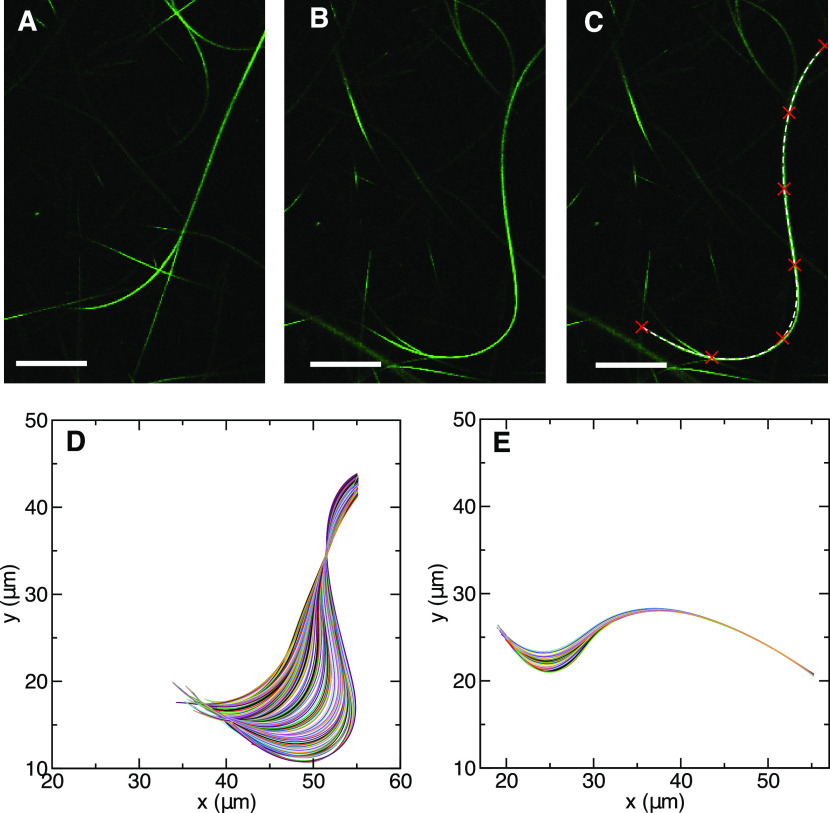
Bending of a microtubule bundle under the action of kinesin clusters. (A, B) Buckling MT bundle over time. Scale bar: 25 μm. (C) Fitting procedure, shown on the bundle from panel (B). The bundle is fitted with a clothoid spline with *N*_p_ = 7 points. (D) Fitted contour of the bundle in panels (A) and (B) over time. (E) Fitted contour of a different bundle over time.

We fitted each contour with clothoid splines (piecewise Euler spirals) to avoid the amplification of noise when calculating the higher derivatives of the bundle shape function. An example of a fit is shown in Figure 2E. The signed curvature *K* of a bundle at a given time was parameterized at *N*_p_ = 7 values along the contour length and interpolated linearly in between. For each set of curvatures, the theoretical shape was calculated with two-fold numerical integration of the interpolated curvature. Finally, the curvature parameters were fitted with a nonlinear least-squares method, using the minimizer nmsimplex2rand from GNU Scientific Library (GSL). Increasing the number of fit parameters to *N*_p_ = 10 led to no visible further improvement of the fit quality.

**Figure 3:**
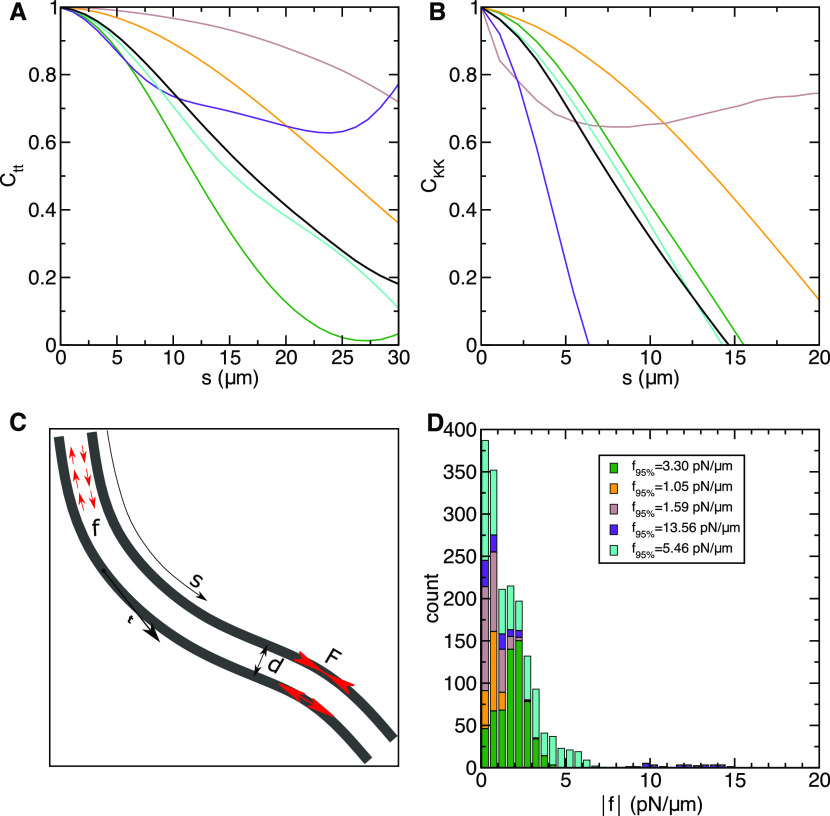
(A) Tangent–tangent correlation *C*_tt_ and (B) curvature correlation *C*_KK_ functions of the individual bundles. Each color represents the results of one bundle and the black line the average. (C) Schematic view of the bundle, represented as a pair of parallel filaments. *f*(*s*) represents the shear force density per unit length and *F*(*s*) the total shear force as a function of the arc length *s*. (D) Force density distribution of all combined experimental observations over time. The insets show the 95th percentile of force distribution for each bundle.

On the interpolated contours, we calculated the tangent–tangent correlation functions (Figure 3A) of each bundle as
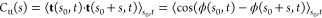
1where **t** denotes the tangent vector of a contour and ϕ its angle relative to the horizontal axis (Figure 3C). The averaging is carried out both along the contour length (*s*_0_) and over video frames at different times *t*.

Likewise, the curvature–curvature correlation function (Figure 3B) is determined as
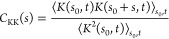
2

## 3 Results and Discussion

### 3.1 Bending Dynamics of Individual Bundles

Using our assembling procedure (see the Experimental Section for details), microtubules polymerized in the active solution and formed a network of active bundles. The bundles were composed of bunches of merged single Taxol-stabilized microtubules that are much shorter than the bundles themselves (average length 17 ± 8 μm) and that spontaneously assemble into a hierarchically higher structure due to depletion forces exerted by the depletant PEG added to the solution.^27^ Namely, when PEG macroparticles are mixed with rod-like particles like microtubules, the PEG induces the phase separation of these filaments to a delimited volume, obtaining microtubules organized into long bundles.^9^ When kinesin clusters are mixed in the same solution, they bind at least to two microtubules due to their multimeric arrangement and cross-link them. Upon addition of ATP, the motors in the clusters “walk” along the filaments exerting contraction and extension forces on the microtubules depending on the filament polarity.^8^ We diluted the mixture to an extent that allows the visualization of some individual bundles. The characterization of their active motion in the bulk can help to better understand the dynamics at a smaller scale that leads to the emergent behavior of active biopolymer networks. The length of the single bundles ranged from 100 to 300 μm. We observed a variety of dynamics that includes continuous bending, buckling, merging through sliding, and disassembly due to the action of the motors. Similar behavior has been observed in the past in systems assembled with shorter microtubules.^28^ These active systems are fundamentally different from their passive counterparts.^29,30^

We are interested in the bending capacity of the bundles due to the activity of the microtubule–motor complex (Figure 2A,B). For this purpose, the motion of the individual bundle has been tracked over time using a custom-made algorithm (see the Experimental Section for details). Although the bundles are composed by numerous filaments moving along the structure under the action of the motors, they are held together by the cross-linking motors and the depletion force exerted by the PEG. Thus, for the tracking, each bundle has been approximated by its centerline (Figure 2C) that corresponded to the fitting function described in the Experimental Section and in the Supporting Information.

The deformation resistance of the system can be estimated by calculating the flexural rigidity *EI*, a characteristic mechanical property of biopolymers. We have formed bundles of microtubules using PEG and added kinesin. To avoid motor activity within the bundles, we did not add ATP. Under these conditions, we determined *EI*_B_ by the image analysis of the thermal fluctuations of the bundles in the bulk.^31^ In a previous study, we have measured *EI* for a single Taxol-stabilized microtubule^32^ and obtained a value of 0.4 × 10^–23^ N m^2^. The value measured here for the bundle was 0.7 × 10^–23^ N m^2^. Interestingly, these values are in a similar range. The flexural rigidity of the bundle *EI*_B_ with weak cross-linking, i.e., held together by PEG, is typically determined as *nEI*, where *n* is the number of microtubules in the bundle and *EI* the flexural rigidity of a single microtubule previously estimated.^32^ Note that with stiffer cross-linkers that simultaneously hold all filaments in the bundle, this can still be described with a worm-like chain model, but with a length-scale-dependent effective stiffness.^33^ Structural X-ray scattering experiments in previous studies^34,35^ found that *n* is determined by the molecular weight and molarity of the depletant PEG mixed in the biopolymer solution. For 20 kDa PEG at 1% (w/v) concentration, it has been observed that the filaments attracted into each bundle are 4.

We characterized the active bundles by analyzing the buckling of the structures due to the motor proteins. Figure 2A,B shows the starting and ending configuration of a bending single bundle. The movement was tracked and the overlap of the filament movement over time can be seen in Figure 2D. By dividing a continuous bundle of length *L* into segments of smaller arc length *s*, the tangent angles θ between these segments can be calculated. As a single bundle continuously bends and buckles over time, we can find a characteristic length over which the tangent–tangent correlation *C*_t__t_(*s*) decays to 50% of its value (Figure 3A). This can be used as a measure of the activity of buckling bundles. We can observe that this characteristic distance varies in the different cases between 12 and 25 μm. To the strong change in the curvature of the bundle depicted in Figure 2B corresponds a characteristic length of 12 μm. However, we could observe also bundles that are already curved and do not show a relevant curvature change over time. In these cases, the evaluation of the characteristic length using the tangent–tangent correlation method is not feasible (Figure 2E).

These different behaviors and configurations can be attributed to the distribution and arrangement of the motor clusters within the microtubule bundles. The filaments are randomly distributed within the bundles in terms of polarity and when antiparallel oriented, they are displaced under the action of the motors. Then, we can observe a strong change in the conformation over time. However, the microtubules within these bundles are all interconnected by high density of motors that act simultaneously on more than two filaments. Under this configuration, the motors can experience high force loads due to filaments they are connected to. This can reduce the stepping speed of the motors and possibly induce them to stall.

By analyzing the curvature in the different bundles over time, we can observe an interesting similarity. The curvature correlation varies in a small range between 3.7 and 13.8 μm revealing that the bending of different microtubule bundles occurs over a similar length scale, although the bundles across multiple samples have heterogeneous lengths. This may be due to the uniform distribution of the motors in the mixture and the average length of the microtubules. These two parameters are independent of the length of the bundles. The curvature of the structure and its variation can also provide precious information related to the forces that the motors exert during their stepping along the microtubules. The bending activity is the result of this force. A direct estimation of these forces *F* is not accessible in our setup.

To reconstruct the force density, we propose a simple model of an active bundle that is bent by a combination of external and internal bending moments^36^
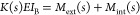
3where *M*_ext_ is the contribution of external torques and torques resulting from external forces and vanishes in a free bundle and *M*_int_ is the internal torque. In a simple bundle consisting of two filaments at distance *d*, it is determined as *M*_int_ = *Fd*, where *F* is the shear force between the filaments, produced by motor proteins (Figure 3C). The density of motor forces per unit length *f* corresponds to the derivative of the force in the filament, *f* = d*F*/d*s*. A similar derivation is valid with more than 2 filaments in the bundle when the shear forces become additive.

If we assume a constant effective distance between filaments *d*, the force density becomes directly proportional to the derivative of the curvature
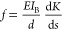
4We used eq 4 to determine the distribution of force densities in experimentally observed bundles. For the distance *d*, we used the average size of the kinesin-1 cluster separating microtubules within a bundle, *d* = 75 nm. Numerically, we determined the curvature derivative as Δ*K*/Δ*s*, where Δ*s* spans between two nodes of the fitted spline (see Section 2.4).

The distribution of force densities in 5 different bundles, each over several time steps, is shown in Figure 3D. The distribution of the force density value in all cases covers similar intervals, mainly ranging between 0 and 5 pN/μm. As 5 pN roughly corresponds to the stall force of a kinesin motor, we conclude that most bundles have a density of active, force generating, motors of about 1 per micrometer length. This roughly correspond to our experimental observation (see Movies S1, S2, and S3). Naturally, motor complexes that do not contribute to the shear force, for example, because they are running on two microtubules with parallel polarity, are not included in this estimate.

We also estimated the maximum force density as the 95th percentile of the distribution. The maximum force densities vary between bundles within an interval from 1.05 to 13.56 pN/μm. The highest densities correspond to about 1 force generating motor complex per 300 nm length. The variability between different bundles likely reflects their composition, e.g., the number of antiparallel microtubules.

### 3.2 Kinesin Clusters Embedded in Microtubule Networks

The dynamics of the individual bundles shown above is the result of the collective motion of the kinesin-1 motors in a multimeric arrangement acting simultaneously on more than one filament within a single bundle. We were interested in characterizing the features of the motor motion at the single cluster level. This can help to understand how clusters of kinesin lead to the bundle deformations observed in this study.

For this purpose, we injected the nondiluted active mixture in the experimental channel. In this way, we obtained both bundles adhering nonspecifically on the glass substrate and floating bundles interacting through the motors with the fixed ones. For clustering the kinesin motors, we used fluorescent streptavidin and it allowed to visualize motor proteins moving between these free and adhering bundles. The fluorescent streptavidin enabled to trace the movement of motors using a custom-made tracking algorithm (see Section 2.3 for details). Motor movements were visible also in floating bundles in the bulk that poorly interacted with the adhering ones. However, we limited our analysis to the motor traces in bundles without visible lateral motion to avoid the need to decompose the observed velocity into motor and bundle motion.

Following this strategy, we tracked *N* = 1128 kinesin motor clusters. Interestingly, *N*_R_ = 80 of these motors, representing a fraction of 7%, showed a direction reversal during their motion along the path (see Movie S3). We measured the run length of the motors in the two populations (nonreversing and reversing) as the sum of the absolute value of all of the unidirectional motion segments *d*_*i*_ they travel within the bundles before finally stalling or detaching and escaping from the visible field (see Movies S1 and S2).

**Figure 4:**
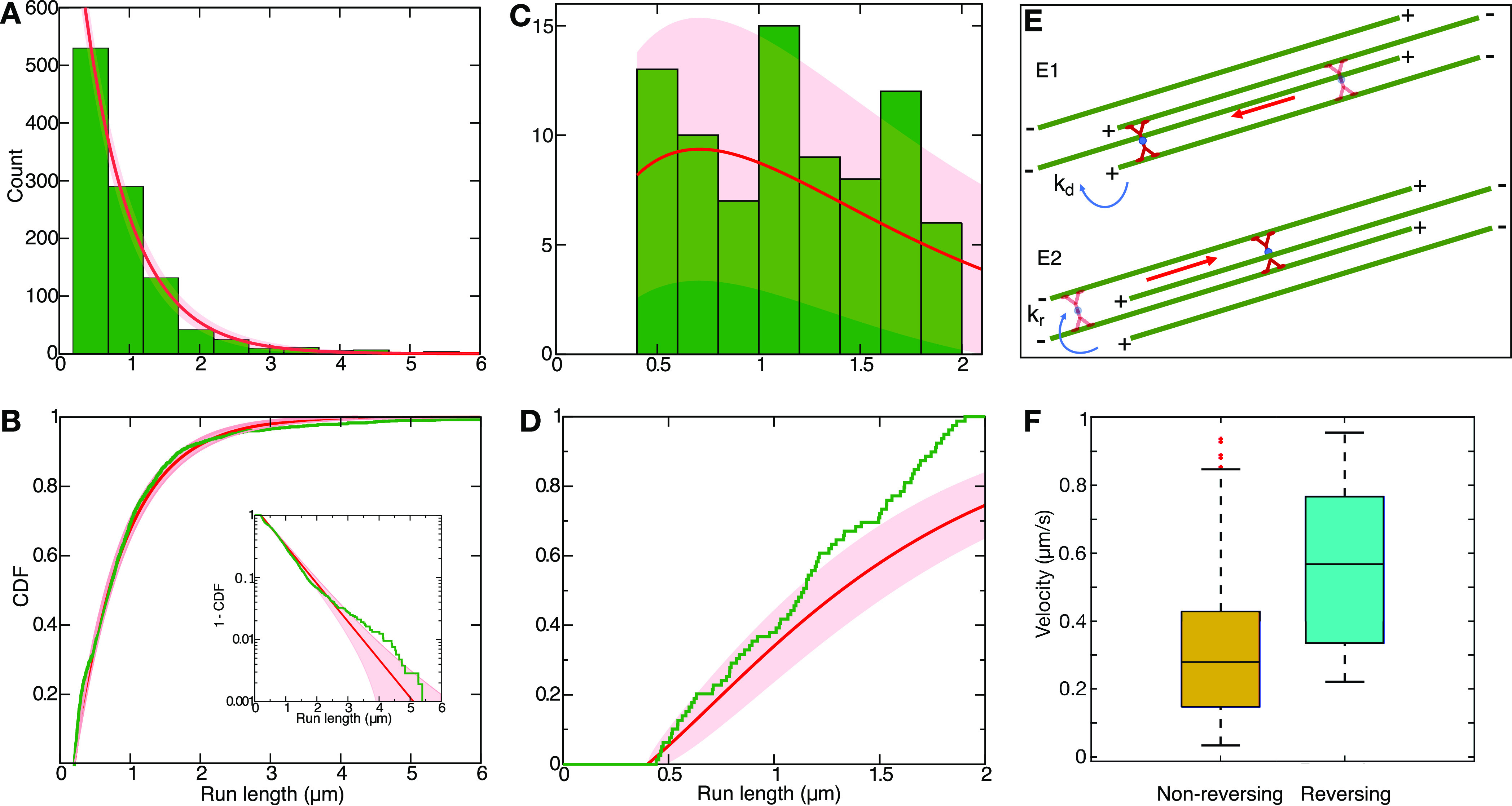
Probability (A) and cumulative distribution function (B) of kinesin-1 motor clusters moving along bundled microtubules without changing their directions (*N* = 1048). The inset in (B) shows the log-linear plot of the complementary CDF. (C, D) Probability distribution and cumulative distribution of those motor clusters that reversed their direction while walking along bundled microtubules (*N* = 80). The experimental distributions in panels (A–D) are shown in green and the theoretical curves (eqs 5, 6, 8, and 9) in red. The 95% confidence interval is represented by the shaded area. (E) Schematic view of a kinesin cluster traveling toward the plus end of a pair of microtubules in the bundle. With rate *k*_d_, the motor detaches from the bundle (either spontaneously or by reaching the end of a microtubule). With rate *k*_*r*_, it switches to microtubules of opposite polarity inside the bundle. (F) Box plot of the velocity of kinesin-1 motor clusters of the two populations, namely, nonreversing and reversing motor clusters.

Figure 4 shows the probability distribution function and the cumulative distribution function of the run length for the two motor populations, which we called nonreversing and reversing motors. We can observe that the run lengths of the nonreversing population are exponentially distributed with an average λ_nr_ = 0.68 ± 0.02 μm (Figure 4A,B). The distribution of run lengths of the reversing population shows a different trend compared to the one described above for the nonreversing motors. The shortest run length measured about 0.4 μm, as it is composed of two motion segments of at least 200 nm each, and the largest one measured about 1.9 μm. Interestingly, all of the run lengths within this interval have similar probabilities to be traveled by the motor clusters. The cumulative distribution presents a linear trend (Figure 4C,D), and the mean value of the run length of the reversing motors is λ_r_ = 0.95 ± 0.05 μm, 50% higher than the nonreversing population.

To understand this difference, we propose a simple theoretical model that describes the detachment and reversal of motors as stochastic processes. We propose that the motor dynamics can be described with a Markovian model in which each motor detaches randomly with the rate *k*_d_ and reverses its direction by switching to a microtubule of opposite polarity with the rate *k*_r_. In this model, the fraction of motors that will reverse their direction at least once before detaching will be η_r_ = *k*_r_/(*k*_r_ + *k*_d_). The probability density function that a motor will have no reversal and will detach after a lifetime *t* is given as *P*(*t*) = exp(−(*k*_d_ + *k*_r_)*t*)*k*_d_. Among motors that show no reversal, the distribution of lifetimes is *P*(*t*) divided by the fraction of motors that show no reversal, 1 – η_r_. If we assume that an attached motor moves with a constant velocity *v*, the observed probability density function for the run length *l* is
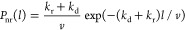
5The cumulative density function (CDF), giving the probability that the run length of a motor among those that show no reversal is shorter than *l*, is obtained by integration
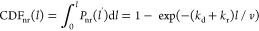
6The probability that a motor shows at least one reversal and detaches after a lifetime *t* can be calculated as
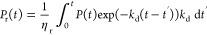
7Converted to the total run length *l*, the distribution is
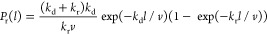
8Again, it can be integrated to obtain the cumulative distribution
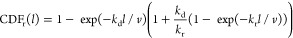
9The distributions obtained above can be compared with the experimental ones. Note that we determine the rates just from the two observed parameters: η = 0.07 and *v*/(*k*_d_ + *k*_r_) = λ_nr_ = 0.68 μm and do not use any fitting parameters. The only adaptation is that the cumulative distributions are stretched to take into account the minimum detectable distance (0.2 μm without reversal and 0.4 μm with reversal). The distributions show a good agreement with the experiments (Figure 4), supporting the model in which motors randomly detach or switch filaments. The mean run length obtained from the distribution *P*_r_(*l*) is λ_r_ = 1.4 μm. If we take into account the fact that run lengths below 0.4 μm are undetected or discarded in the experiment and calculate the mean of the sections above this threshold, we obtain λ̅_r_ = 1.15 μm, close to the experimentally observed value. In comparison with theoretical prediction, events in the interval between 1.5 and 2 μm are overrepresented at the cost of run lengths longer than 2 μm. This causes that the theoretical model does not fit as well as for data shorter than 1.5 μm. In the Markovian picture, the longer run length among motors that show a reversal is not surprising and can also be interpreted as a selection bias: all motors have the same a priori distribution of run lengths, but those with longer runs have a higher likelihood of directionality reversal.

From the data and the model, we can also convert the reversal rate into a distance Λ_r_ = *v*/*k*_r_ = λ_nr_/η_r_ = 9.7 μm. Its inverse Λ_r_^–1^ gives the likelihood of direction reversal per unit length traveled, regardless of detachment events. The reversal distance Λ_r_ is close to the curvature correlation length. This suggests that although the motor run length is much shorter, their reversal could be governed by changes in the microtubule polarity distribution in the bundles, which also determines the active bending.

In a previous study, it was already observed that kinesin-1 motors reverse their direction during their movement along the microtubule bundles aggregated by PEG.^37^ As in our case, the filaments in the bundles are randomly oriented and the motors are likely able to bind to nearby filaments with an opposed polarity and reverse their direction. Also for the previous observations, the run length of the reversing motors was longer than that of the nonreversing motors. However, the measured run length in ref (37) is 2 and 3 times higher than the run length of nonreversing and reversing motors in our study, respectively. We believe that the difference is due to our experimental setup, namely, the motors arranged in clusters and used at high density in the bundles. Each motor in the cluster is likely associated to microtubules that are simultaneously displaced by other motors. In this way, the motors can indirectly interfere with each other and the motor stepping can be slowed down or paused. This can briefly block the movement of the other motors, resulting in reduced processivity compared to the single motor case.^37^ We propose that the shorter run length and the slower motor stepping cause also a lower proportion of clusters that reversed direction, namely, 7% compared to 34% in the previous study.

The velocity of the two populations during their movement on the microtubules can be calculated considering the run length covered by each cluster over the time the motors spend walking without possible short pauses. As can be seen in Figure 4F, the median of the velocity of the motors that walk in one direction without reversing is *v*_nr_ = 0.28 ± 0.15 μm/s, whereas the motors reversing their direction have a median value of *v*_r_ = 0.57 ± 0.19 μm/s. However, there is no statistically significant difference between the velocity of the two populations. This is consistent with the picture that the switching is a random process independent of kinesin motility before and after the switch.

The mutual motor interference leads to brief pauses and accelerated detachment from the filament. In the 7% of the motor cluster that reverse the direction, we can hypothesize that all of the motors in the cluster simultaneously detach from their microtubules and likely associate with some with opposite direction that offer them additional free path to move.

## 4 Conclusions

In this study, we characterize the dynamics of an active network made of microtubules and kinesin motors at the single bundle level. In this way, we could eliminate the effects of the interaction between adjacent bundles that dominate in high-density active nematics. Our work thus bridges the gap between the single-molecule studies on motor proteins and experiments with large-scale active networks. We demonstrated the bending motion of randomly assembled individual bundles, driven by kinesin-1 motor clusters, and correlated it with the motility of motor clusters. We showed that a separation of length scales takes place: while the typical run length of kinesin clusters is below 1 μm, the actively induced curvature has a correlation length of ∼10 μm. We propose that the bending dynamics is determined by the distribution of microtubule polarities in the bundle because kinesin clusters only exert a significant force between antiparallel microtubules. We also observed that a small kinesin cluster population exists that shows a reversal in the direction of motion, for instance, due to the detachment from a filament and the reattachement to another microtubule with opposite polarity in the bundle. By elucidating the microtubule bundle dynamics, our study contributes to the understanding of active bundles that accomplish many vital functions in the cell, for instance, the formation of mitotic spindles.^38^ Furthermore, the understanding of isolated active bundles is a prerequisite for studying synthetic active networks with a dynamics frequently driven by extensile microtubule bundles.^8,14,15^

## Supporting Information Available

Supporting material and methods and schematics of the experimental chamber for microscopy of active microtubule bundles (Figure S1) (PDF)
Tracked movement of a motor cluster (Movie S1) (MP4).
Tracked movement of a motor cluster that completely detaches from the associated microtubule bundle (Movie S2) (MP4)
Forward and backward movement of a motor cluster (Movie S3) (MP4)
