# Supplementary material for: Active Bending of Disordered Microtubule Bundles by Kinesin Motors
Source: ACS Omega. 2022 Nov 18;7(48):43820–8. doi: 10.1021/acsomega.2c04958 (PMC9730755; doi:10.1021/acsomega.2c04958)
Supplement: Supplementary file 1 — ao2c04958_si_001.pdf [file ao2c04958_si_001.pdf]

Supporting Information
Active bending of disordered microtubule bundles by kinesin motors

Vahid Nasirimarekani,[†,‖] Smrithika Subramani,[†,‡,‖] Sebastian Herzog,[¶,†] Andrej Vilfan,[†,§] and Isabella Guido[*,†]

†Max Planck Institute for Dynamics and Self-Organization (MPIDS), Am Fassberg 17, 37077 Göttingen, Germany

‡University of Wisconsin-Milwaukee, Department of Physics, 3135 N Maryland Ave, Milwaukee, WI 53211, USA

¶University of Göttingen, Department for Computational Neuroscience, Third Institute of Physics - Biophysics, Friedrich-Hund-Platz 1, 37077 Göttingen, Germany

§Jožef Stefan Institute, Jamova 39, 1000 Ljubljana, Slovenia

‖These authors contributed equally

E-mail: isabella.guido@ds.mpg.de

Materials and Methods

Tracking

The tracking consists of two steps, a segmentation of the individual motors and the tracking of the motors over time. The segmentation routine was developed in house based on Fiji[1] and the tracking was done with TrackMate[2]. Both routines were implemented as ImageJ macros and applied to each of the experimentally collected cases. The segmentation

consists of several steps to extract masks from the images that remove everything except the motors to be tracked.

The segmentation consists of several steps as shown in algorithm to extract masks from the images which remove everything except the motors to be tracked. The application of

---

Algorithm 1: Segmentation algorithm

Data: Raw images
Result: Segemented images
1. Removal of the minimum intensities over the whole image stack;
2. Background subtraction;
3. Gaussian blur to form the masks;
4. Thresholding to form the final masks;
5. Further particle analysis to allow only masks that have a circularity of $[0.9, 1.0]$ and a size of $[9, 100]$ pixels;
6. Application of the masks to the individual images from the stack;

---

algorithm provides segmented images which are then directly used as input in TrackMate[2]. In TrackMate the parameters for the maximal linking distance was set to 2.0 pixel, with a maximal gap closing distance of 2.0 pixel and no gap closing.

Code for segmentation

```
run("Close All");
run("Clear Results");

dirPath="<PathToRawData>"
fileName="testmotorwalk18_R3D.dv"
outputPath="<OutPutPath>" + substring(fileName,0,lengthOf(fileName)-3)+".tif"

open(dirPath+fileName);
selectWindow(fileName+" - C=0");
rename("working");

```

```
run("Duplicate...", "title=raw_imgs duplicate");
selectWindow("working");

run("Z Project...", "projection=[Min Intensity]");
imageCalculator("Subtract create stack", "working","MIN_working");
run("Subtract Background...", "rolling=2 sliding stack");
run("Gaussian Blur...", "sigma=1 stack");

setThreshold(5, 65535);
run("Convert to Mask", "method=Default background=Dark");
rename("threshold");

selectWindow("working");

for (i=1; i<=nSlices; i++) {
        selectWindow("threshold");
    setSlice(i);
        run("Analyze Particles...", "size=9-100 pixel circularity=0.90-1.00
         ↪  display clear add slice");
        selectWindow("working");
        setSlice(i);
        roiManager("XOR");
        run("Make Inverse");
        run("Cut");
}
run("Select None");
selectWindow("working");
saveAs("Tiff", outputPath);
```

Code for running trackmage

```
\label{alg:track}
from ij import IJ, ImagePlus, ImageStack
import fiji.plugin.trackmate.Settings as Settings
import fiji.plugin.trackmate.Model as Model
import fiji.plugin.trackmate.SelectionModel as SelectionModel
import fiji.plugin.trackmate.TrackMate as TrackMate
import fiji.plugin.trackmate.Logger as Logger
import fiji.plugin.trackmate.detection.DetectorKeys as DetectorKeys
import fiji.plugin.trackmate.detection.DogDetectorFactory as DogDetectorFactory
import fiji.plugin.trackmate. tracking.sparselap.SparseLAPTrackerFactory as SparseLAPTrackerFactory
import fiji.plugin.trackmate. tracking.LAPUtils as LAPUtils
import fiji.plugin.trackmate. visualization.hyperstack.HyperStackDisplayer as HyperStackDisplayer
import fiji.plugin.trackmate. features.FeatureFilter as FeatureFilter
import fiji.plugin.trackmate. features.FeatureAnalyzer as FeatureAnalyzer
import fiji.plugin.trackmate. features.spot.SpotContrastAndSNRAnalyzerFactory as SpotContrastAndSNRAnalyzerFactory
import fiji.plugin.trackmate. action.ExportStatsToIJAction as ExportStatsToIJAction
import fiji.plugin.trackmate. io.TmXmlReader as TmXmlReader
import fiji.plugin.trackmate. action.ExportTracksToXML as ExportTracksToXML
import fiji.plugin.trackmate. io.TmXmlWriter as TmXmlWriter
import fiji.plugin.trackmate. features.ModelFeatureUpdater as ModelFeatureUpdater
```

```
import fiji.plugin.trackmate. features.SpotFeatureCalculator as SpotFeatureCalculator
import fiji.plugin.trackmate. features.spot.SpotContrastAndSNRAnalyzer as SpotContrastAndSNRAnalyzer
import fiji.plugin.trackmate. features.spot.SpotIntensityAnalyzerFactory as SpotIntensityAnalyzerFactory
import fiji.plugin.trackmate. features.track.TrackSpeedStatisticsAnalyzer as TrackSpeedStatisticsAnalyzer
import fiji.plugin.trackmate. util.TMUtils as TMUtils
import sys
import java.io.File as File

imageName = "testmotorwalk18_R3D.tif"
pathToImgs = "<PathtoSegmentedImages>"
inputImage = pathToImgs + imageName

imp = IJ.openImage(inputImage)
model = Model()
model.setLogger(Logger.IJ_LOGGER)

settings = Settings()
settings.setFrom(imp)

# Configure detector
settings.detectorFactory = DogDetectorFactory()
settings.detectorSettings = {
    DetectorKeys.KEY_DO_SUBPIXEL_LOCALIZATION: True,
    DetectorKeys.KEY_RADIUS: 1.0,
```

```python
    DetectorKeys.KEY_TARGET_CHANNEL: 1,
    DetectorKeys.KEY_THRESHOLD: 1.,
    DetectorKeys.KEY_DO_MEDIAN_FILTERING: False,
}

# Configure tracker
settings.trackerFactory = SparseLAPTrackerFactory()
settings.trackerSettings = LAPUtils.getDefaultLAPSettingsMap()
settings.trackerSettings['LINKING_MAX_DISTANCE'] = 2.0
settings.trackerSettings['GAP_CLOSING_MAX_DISTANCE'] = 2.0
settings.trackerSettings['MAX_FRAME_GAP'] = 0
settings.addSpotAnalyzerFactory( SpotIntensityAnalyzerFactory() )
settings.addSpotAnalyzerFactory( SpotContrastAndSNRAnalyzerFactory() )
settings.addTrackAnalyzer(TrackSpeedStatisticsAnalyzer())
settings.initialSpotFilterValue = 1

trackmate = TrackMate(model, settings)

ok = trackmate.checkInput()
if not ok:
    sys.exit(str(trackmate.getErrorMessage()))

ok = trackmate.process()
if not ok:
    sys.exit(str(trackmate.getErrorMessage()))

model.getLogger().log('Found ' + str(model.getTrackModel().nTracks(True)) + ' tracks.')
```

```python

selectionModel = SelectionModel(model)
displayer = HyperStackDisplayer(model, selectionModel, imp)
displayer.render()
displayer.refresh()

fm = model.getFeatureModel()

for id in model.getTrackModel().trackIDs(True):
    v = fm.getTrackFeature(id, 'TRACK_MEAN_SPEED')
    model.getLogger().log('')
    model.getLogger().log('Track ' + str(id) + ': mean velocity = ' + str(v) +' ' + model.getSpaceUnits() + '/' + model.getTimeUnits())

    track = model.getTrackModel().trackSpots(id)
    for spot in track:
        sid = spot.ID()
        # Fetch spot features directly from spot.
        x = spot.getFeature('POSITION_X')
        y = spot.getFeature('POSITION_Y')
        t = spot.getFeature('FRAME')
        q = spot.getFeature('QUALITY')
        snr = spot.getFeature('SNR')
        mean = spot.getFeature('MEAN_INTENSITY')
        model.getLogger().log('\tspot ID = ' + str(sid) + ': x=' + str(x) + ', y=' + str(y) +', t=' + str(t) + ', q=' + str(q) + ', snr=' + str(snr) + ', mean = ' + str(mean))

```

```
outFile = File(pathToImgs, "tracks_" + imageName.split(".")[0] + ".xml")
ExportTracksToXML.export(model, settings, outFile)
```

For each experimental case, both macros are executed in succession. The code is publicly available.

Microfluidics Channel

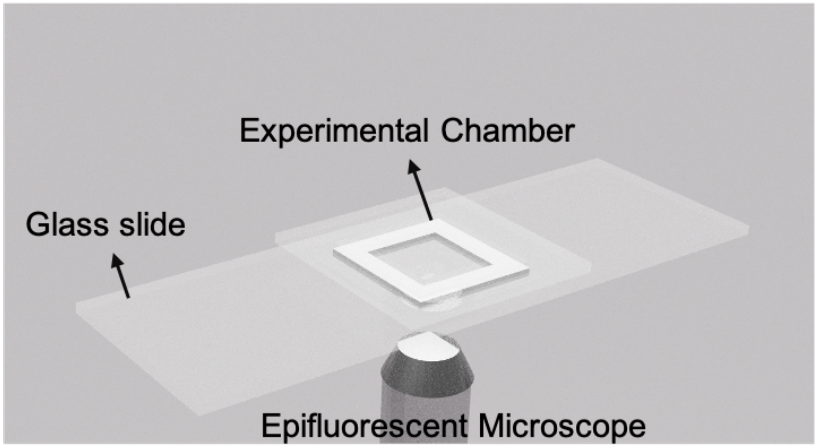

Figure S 1 Schematics of the experimental chamber for microscopy of active microtubule bundles.

Supplementary Movies

In all the movies, one motor cluster is tracked over time and the different behaviours are shown. The cluster is marked by a blue dot and the the trajectory by a blue line. The frame rate is 13 fps.

Movie S1

The movie shows the tracked movement of a motor cluster that walks forward, stops and stalls after covering a certain distance.

Movie S2

The movie shows the tracked movement of a motor cluster that completely detaches from the associated microtubule bundle after walking along it.

Movie S3

The movie shows the forward and backward movement of a motor cluster.

Notes and references

(1) Schindelin, J.; Arganda-Carreras, I.; Frise, E.; Kaynig, V.; Longair, M.; Pietzsch, T.; Preibisch, S.; Rueden, C.; Saalfeld, S.; Schmid, B., et al. Fiji: an open-source platform for biological-image (IN) analysis. *Nat. Methods* **2012**, *9*, 676–682.

(2) Tinevez, J.-Y.; Perry, N.; Schindelin, J.; Hoopes, G. M.; Reynolds, G. D.; Laplantine, E.; Bednarek, S. Y.; Shorte, S. L.; Eliceiri, K. W. TrackMate: An open and extensible platform for single-particle tracking. *Methods* **2017**, *115*, 80–90, Image (IN) Processing for Biologists.
